# Effects of tumour necrosis factor on cardiovascular disease and cancer: A two-sample Mendelian randomization study

**DOI:** 10.1016/j.ebiom.2020.102956

**Published:** 2020-08-14

**Authors:** Shuai Yuan, Paul Carter, Maria Bruzelius, Mathew Vithayathil, Siddhartha Kar, Amy M. Mason, Ang Lin, Stephen Burgess, Susanna C. Larsson

**Affiliations:** a Unit of Cardiovascular and Nutritional Epidemiology, Institute of Environmental Medicine, Karolinska Institutet, Stockholm 17177, Sweden; b Department of Surgical Sciences, Uppsala University, Uppsala, Sweden; c Department of Public Health and Primary Care, University of Cambridge, Cambridge, United Kingdom; d Coagulation Unit, Department of Hematology, Karolinska University Hospital, Stockholm, Sweden; e Department of Medicine Solna, Karolinska Institutet, Stockholm, Sweden; f MRC Cancer Unit, University of Cambridge, Cambridge, United Kingdom; g MRC Integrative Epidemiology Unit, Bristol Medical School, University of Bristol, Bristol, United Kingdom; h British Heart Foundation Cardiovascular Epidemiology Unit, Department of Public Health and Primary Care, University of Cambridge, Cambridge, United Kingdom; i National Institute for Health Research Cambridge Biomedical Research Centre, University of Cambridge and Cambridge University Hospitals, Cambridge, United Kingdom; j Department of Medicine Solna, Division of Immunology and Allergy, Karolinska Institutet, Stockholm, Sweden; k Center for Molecular Medicine, Karolinska Institutet, Stockholm, Sweden; l MRC Biostatistics Unit, University of Cambridge, Cambridge, United Kingdom

**Keywords:** Cancer, Cardiovascular disease, Tumour necrosis factor

## Abstract

**Background:**

Tumour necrosis factor (TNF) inhibitors are used in the treatment of certain autoimmune diseases but given the role of TNF in tumour biology and atherosclerosis, such therapies may influence the risk of cancer and cardiovascular disease. We conducted a Mendelian randomization study to explore whether TNF levels are causally related to cardiovascular disease and cancer.

**Methods:**

Single-nucleotide polymorphisms associated with TNF levels at genome-wide significance were identified from a genome-wide association study of 30 912 European-ancestry individuals. Three TNF-associated single-nucleotide polymorphisms associated with higher risk of autoimmune diseases were used as instrumental variables. Summary-level data for 14 cardiovascular diseases, overall cancer and 14 site-specific cancers were obtained from UK Biobank and consortia.

**Findings:**

Genetically-predicted TNF levels were positively associated with coronary artery disease (odds ratio (OR) 2.25; 95% confidence interval (CI) 1.50, 3.37) and ischaemic stroke (OR 2.27; 95% CI 1.50, 3.43), and inversely associated with overall cancer (OR 0.54; 95% CI 0.42, 0.69), breast cancer (OR 0.51; 95% CI 0.39, 0.67), and colorectal cancer (OR 0.20; 95% CI 0.09, 0.45). There were suggestive associations of TNF with venous thromboembolism (OR 2.18; 95% CI 1.32, 3.59), endometrial cancer (OR 0.25; 95% CI 0.07, 0.94), and lung cancer (OR 0.45; 95% CI 0.21, 0.94).

**Interpretation:**

This study found evidence of causal associations of increased TNF levels with higher risk of common cardiovascular diseases and lower risk of overall and certain cancers.

**Research in context**

**Evidence before this study**

Tumour necrosis factor (TNF) is a pro-inflammatory cytokine secreted primarily by immune cells. It is involved in a broad range of both homoeostatic and pathophysiological processes, such as immunity, inflammation, cell proliferation, apoptosis and lipid metabolism. As such, anti-TNF agents have become cornerstone in the treatment of autoimmune inflammatory conditions such as rheumatoid arthritis and inflammatory bowel disease. However, the potential therapeutic, or even deleterious, effects of targeting TNF in other inflammatory conditions, such as cardiovascular disease and cancer remains equivocal. Mendelian randomization (MR) is an epidemiological approach using genetic variants as instrumental variables for an exposure to strengthen the causal inference in an exposure-outcome association by reducing residual confounding and reverse causality.

**Added value of this study**

In the present MR study, we provided the first causal evidence of positive associations of TNF levels with atherothrombotic disease (coronary artery disease and ischaemic stroke) and venous thromboembolism. Furthermore, we revealed inverse associations of TNF levels with risk of overall cancer and several site-specific cancers (colorectal, breast, endometrial, and lung cancers). We confirmed that higher TNF levels were strongly associated with established TNF-driven diseases (rheumatoid arthritis and inflammatory bowel disease) which added strong support to the validity of the genetic instrument used and the reliability of our findings.

**Implications of the all the available evidence**

This study reveals evidence of causal associations of increased TNF levels with higher risk of common cardiovascular diseases and lower risk of overall and certain cancers. These results may inform decisions concerning potential benefits and risks of TNF inhibitor therapy. In detail, clinicians need to assess potential increased cancer risk derived from anti-TNF therapy usage especially amongst individuals with inherited or acquired high risk of cancer, and in addition, may use anti-TNF medicine as a potential prevention approach for people with excessive cardiovascular risk and a potential treatment strategy for patients with impaired cardiovascular condition. The study also indicates that randomized controlled trials are warranted to verify our findings and comprehensively evaluate the benefits and risks of anti-TNF therapy in populations with different health conditions.

Alt-text: Unlabelled box

## 1 Introduction

Tumour necrosis factor (TNF) is a pro-inflammatory cytokine secreted primarily by immune cells. It is involved in a broad range of both homoeostatic and pathophysiological processes, such as immunity, inflammation, cell proliferation, apoptosis and lipid metabolism [1], [2], [3]. As such, anti-TNF agents have become cornerstone in the treatment of autoimmune inflammatory conditions such as rheumatoid arthritis and inflammatory bowel disease. However, the potential therapeutic, or even deleterious, effects of targeting TNF in other inflammatory conditions, such as cardiovascular disease (CVD) and cancer remains equivocal.

Atherosclerosis is a chronic inflammatory disease of the arterial wall, driven by immune cells and cytokines at all stages, and, TNF-deficient mice have reduced plaque size [4]. This is likely of importance in humans as TNF levels post-myocardial infarction are a strong predictor of recurrent events [5]. Furthermore, multiple observational studies have shown that TNF inhibition reduces atherosclerosis and cardiovascular events when administered to patients with rheumatoid arthritis [6]. Whether this benefit is also conferred in the general population, rather than patients suffering from conditions characterized by enhanced TNF activity, is poorly understood. Similarly, the role of TNF in heart failure remains equivocal. Although epidemiologically, TNF levels are predictive of heart failure mortality [7], a clinical trial in heart failure patients observed a higher hospitalization rate in the group receiving 10 mg/kg infliximab (anti-TNF) compared with the placebo group [8]. The potentially causal role of TNF in heart failure, atherosclerosis in a range of vascular beds and other cardiovascular diseases therefore need to be investigated.

Cancer is characterized by uncontrolled cell proliferation and survival. As a pro-inflammatory cytokine, TNF can promote all stages of carcinogenesis including survival, angiogenesis, and metastasis. TNF levels are raised in multiple cancer types, are reduced by chemotherapy and the reduction is associated with patient outcomes [9]. TNF inhibition may therefore be a potential cancer therapy. However, there have been multiple reports of increased risk of certain malignancies such as squamous cell cancer [10] in patients treated with anti-TNF agents. This may relate to the paradoxical tumour-suppressive effects of TNF, such as cytotoxicity. Thus, TNF and anti-TNF therapies may both have carcinogenic benefits and risks in different cancer types and the causal role of the cytokine in the development of a wide range of site-specific cancers warrants further evaluation.

Utilizing genetic variants as instrumental variables for an exposure (e.g., TNF levels), Mendelian randomization (MR) can improve the causal inference of an exposure-outcome association [11]. It minimizes potential methodological limitations, such as confounding and reverse causality. The rationale for diminished bias in MR studies is that genetic variants are randomly assorted and fixed at conception and therefore largely independent of confounders and cannot be modified by disease development [11].

Here, we aimed to evaluate the CVDs and cancers that are causally associated with TNF levels and which could be targeted with TNF-modifying therapies. We conducted a two-sample Mendelian randomization study to explore the associations of genetically predicted TNF levels with risk of 14 CVDs, overall cancer, and 14 site-specific cancers. To validate the instrumental variables, we assessed whether genetically predicted TNF levels were associated with higher risk of rheumatoid arthritis and inflammatory bowel disease.

## 2 Methods

### 2.1 Study design

This is a two-sample MR study design based on summary-level data. An MR analysis depends on the assumptions that the genetic variants: [1] are strongly associated with the exposure (the relevance assumption); [2] are not associated with confounders of the exposure-outcome relationship (the independence assumption); and [3] have an effect on the outcome through the exposure only and not through any other causal pathway (the exclusion restriction assumption) [11]. This MR study has been approved by the Swedish Ethical Review Authority.

### 2.2 Instrumental variable selection and outcome sources

A meta-analysis of genome-wide association studies (GWASs) of 25 cohorts encompassing 30 912 European-descent individuals identified four single-nucleotide polymorphisms (SNPs) associated with TNF levels at genome-wide significance (*P*<5 × 10^−8^) (Table 1) [12]. To ensure that the relevance assumption is likely to be satisfied, we used rheumatoid arthritis [13] and inflammatory bowel disease [14] as positive controls to select SNPs (**Supplementary Table 1**). A genetic instrument containing rs10744774, rs3184504 and rs7182229 was associated with an expected increased odds of rheumatoid arthritis and inflammatory bowel disease. The TNF-raising allele of rs2857602 was associated with lower odds of these autoimmune diseases and was regarded as an unreliable instrumental variable for TNF. We therefore used three SNPs (rs10744774, rs3184504 and rs7182229) as instrumental variables for TNF levels in the primary analysis; all four SNPs were used in a supplementary analysis. The two SNPs on chromosome 12 (rs10744774 and rs3184504) were in modest linkage disequilibrium (*r*^2^ = 0.18) based on 1000 G reference panel. The genotype associations with TNF levels were adjusted for age^2^, sex, body mass index, and study-specific variables such as genetic principal components and relatedness [12].

**Table 1:** Detailed information of instrumental variables for TNF levels.

rsID	Chr	Position (hg19)	Nearby gene	EA	NEA	EAF	Beta	SE	P	Included in main analysis
rs2857602	6	31,533,378	*LTA*	G	A	0.38	0.032	0.006	3.30 × 10^−12^	No
rs10744774	12	112,090,022	*BRAP*	A	C	0.83	0.044	0.007	6.94 × 10^−11^	Yes
rs3184504	12	111,884,608	*SH2B3*	T	C	0.48	0.030	0.005	3.96 × 10^−10^	Yes
rs7182229	15	58,765,183	*LIPC*	T	G	0.11	0.050	0.009	1.07 × 10^−9^	Yes

Chr indicates chromosome; EA; effect allele; EAF, effect allele frequency; NEA, non-effect allele; SE, standard error; TNF, tumour necrosis factor. Rs2857602 was not included in the main analysis since the TNF-increasing allele was associated with lower odds of rheumatoid arthritis and inflammatory bowel disease.

Fourteen CVDs, overall cancer, and 14 site-specific cancers were included as outcomes in this MR study (Table 2). Summary-level data for outcomes were obtained from UK Biobank [15] and genetic consortia [16], [17], [18], [19], [20], [21], [22]. Rs2857602 was not available in the consortia datasets of coronary artery disease and stroke and was replaced by a proxy (rs2844484, *r*^2^=1). From UK Biobank, we included CVDs and cancers with at least 1000 cases to ensure sufficient statistical power to detect moderate to strong associations. The SNP-outcome associations in UK Biobank and most consortia were adjusted for age, sex, and genetic principal components. Detailed information of included outcomes is displayed in Table 2.

**Table 2:** Characteristics of included studies or consortia of inflammatory diseases, cardiovascular diseases, and cancers.

Outcome	Source	Cases	Controls	Sample size	Population
**Inflammatory disease**					
Rheumatoid arthritis	GARNET consortium	29 880	73 758	103 638	Mix
Inflammatory bowel disease	UK IBD consortium	25 042*	34 915	59 957	European
**Cardiovascular disease**					
Cerebrovascular disease					
Overall stroke	MEGASTROKE consortium	67 162	454 450	521 612	Mix
Overall stroke	UKBB	9652	357 991	367 643	European
Any ischaemic stroke	MEGASTROKE consortium	60 341	NA	NA	Mix
Any ischaemic stroke	UKBB	3554	364 089	367 643	European
Large artery stroke	MEGASTROKE consortium	6688	146 392	153 080	Mix
Small vessel stroke	MEGASTROKE consortium	11 710	192 662	204 372	Mix
Cardioembolic stroke	MEGASTROKE consortium	9006	204 570	213 576	Mix
Intracerebral haemorrhage	UKBB	1064	366 579	367 643	European
Subarachnoid haemorrhage	UKBB	1084	366 559	367 643	European
Heart and valvular disease					
Coronary artery disease	CARDIoGRAMplusC4D consortium	60 801	123 504	184 305	Mix
Coronary artery disease	UKBB	24 531	343 112	367 643	European
Heart failure	UKBB	7382	387 652	395 034	European
Atrial fibrillation	AFGen	65 446	522 000	587 446	Mix
Atrial fibrillation	UKBB	16 945	350 698	367 643	European
Abdominal aortic aneurysm	UKBB	1094	366 549	367 643	European
Aortic valve stenosis	UKBB	2244	365 399	367 643	European
Vessel disease					
Peripheral artery disease	UKBB	3415	364 228	367 643	European
Venous thromboembolism	UKBB	15 602	352 041	367 643	European
**Cancer**					
Bladder cancer	UKBB	2588	365 055	367 643	European
Breast cancer	BCAC	122 977	105 974	228 951	Mix
Breast cancer ER-	BCAC	21 468	NA	NA	Mix
Breast cancer ER+	BCAC	69 501	NA	NA	Mix
Breast cancer	UKBB	13 666	353 977	198 838	European
Cervical cancer	UKBB	1928	365 715	198 838	European
Colorectal cancer	UKBB	5486	362 157	367 643	European
Endometrial cancer	UKBB	1520	366 123	198 838	European
Head-neck cancer	UKBB	1615	366 028	367 643	European
Kidney cancer	UKBB	1310	366 333	367 643	European
Leukaemia	UKBB	1403	366 240	367 643	European
Lung cancer	ILCCO	11 348	15,861	27 209	European
Melanoma	UKBB	4869	362 774	367 643	European
Non-Hodgkin's lymphoma	UKBB	2296	365 347	367 643	European
Ovarian cancer	UKBB	1520	366 123	198 838	European
Ovarian cancer	OCAC	22 406	40 941	63 347	Mix
Overall cancer	UKBB	75 037	292 606	367 643	European
Pancreatic cancer	UKBB	1264	366 379	367 643	European
Prostate cancer	PRACTICAL	79 194	61 112	140 306	European
Prostate cancer	UKBB	7872	359 771	168 748	European

AFGen indicates Atrial Fibrillation Consortium; BCAC, Breast Cancer Association Consortium; CARDIoGRAMplusC4D, Coronary ARtery DIsease Genome wide Replication and Meta-analysis plus The Coronary Artery Disease Genetics; GARNET, Genetics and Allied research in Rheumatic diseases Networking; ILCCO, The International Lung Cancer Consortium; NA, not available; OCAC, The Ovarian Cancer Association Consortium; PRACTICAL, The Prostate Cancer Association Group to Investigate Cancer Associated Alterations in the Genome consortium; UKBB, UK Biobank; UK IBD consortium, UK Inflammatory Bowel Disease Genetics Consortium.

⁎ Includes Crohn's disease and ulcerative colitis.

### 2.3 Pleiotropy assessment

To evaluate whether the exclusion restriction assumption is likely to hold, possible pleiotropic associations of the instrumental variables with other phenotypes were assessed by searching a database of human genotype-phenotype associations (PhenoScanner V2) (http://www.phenoscanner.medschl.cam.ac.uk/). One or more of the SNPs related to TNF were associated with autoimmune diseases (coeliac disease, rheumatoid arthritis, and type 1 diabetes), various immune and blood cells, haemoglobin levels, hypothyroidism, diastolic blood pressure, total and low-density lipoprotein cholesterol, and height (**Supplementary Table 2**).

### 2.4 Statistical analysis

The inverse-variance weighted method with adjustment for correlations amongst the SNPs [23] was used to analyse the associations of TNF with CVD and cancer outcomes in the main analysis. A matrix of correlations amongst used SNPs was added into the traditional inverse-variance weighted model, thereby diminishing the effects of linkage disequilibrium [23]. All odds ratios (ORs) and 95% confidence intervals (CIs) of the outcomes were expressed per one unit increase in natural log of TNF (pg/ml). We calculated the statistical power using a web-tool and results of the power analyses are presented in **Supplementary Table 3** [24]. To account for multiple testing, we deemed associations with *p* values below 1.7 × 10^−3^ (where *p* = 0.05/29 (29 outcomes)) as strong evidence of causal associations. Associations with *p* values below 0.05 but above 1.7 × 10^−3^ were treated as suggestive evidence of associations. All analyses were two-sided and performed using TwoSampleMR and MendelianRandomization packages in R 3.6.0.

### 2.5 Role of funders

The funders had no role in study design, data collection, interpretation, or the decision to submit the work for publication.

## 3 Results

**Fig. 1:**
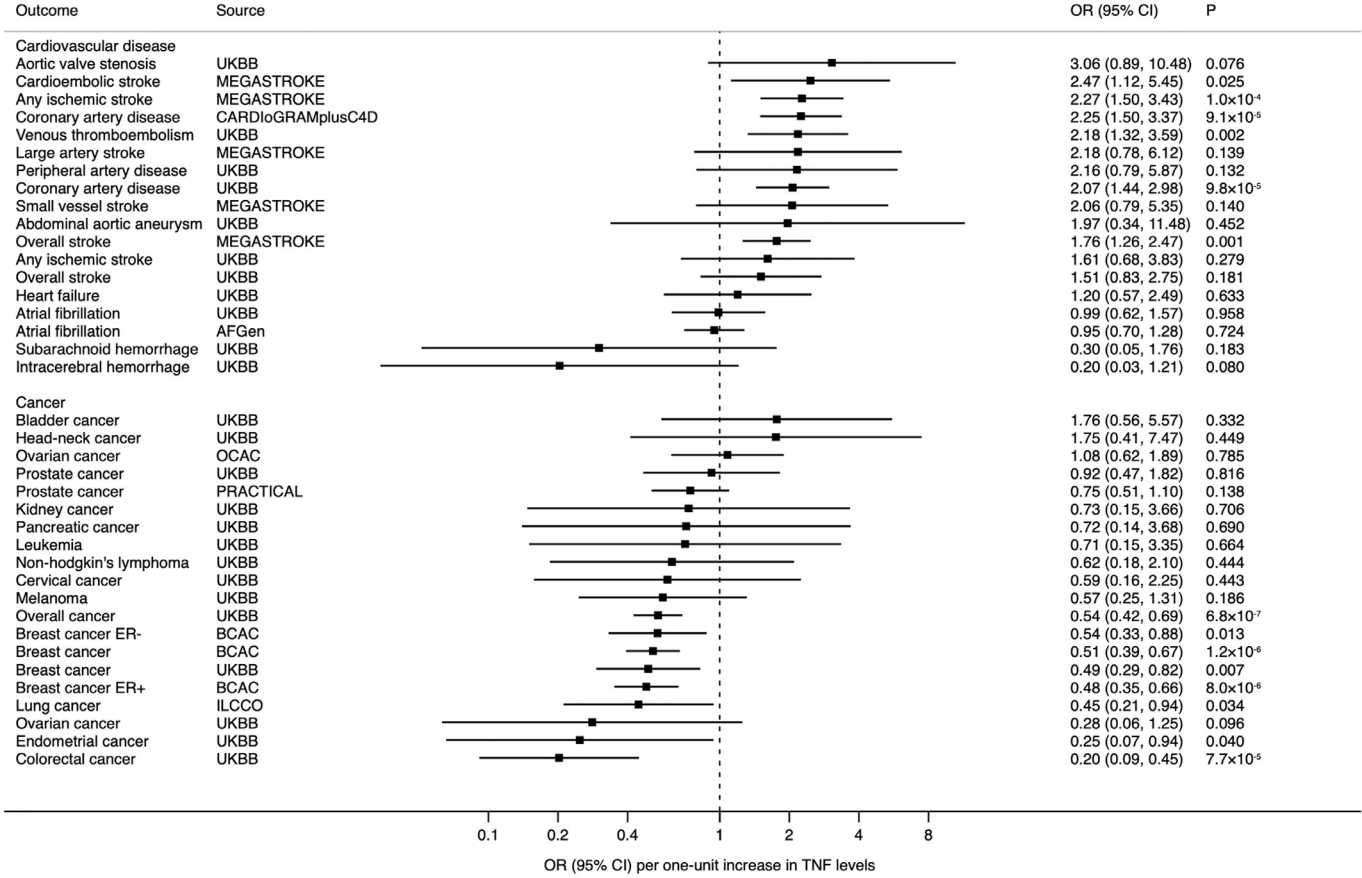
Overview of this MR study, including genetic instrument and data sources used, results, and conclusions. AFGen indicates Atrial Fibrillation Consortium; BCAC, Breast Cancer Association Consortium; Ca, cancer; CAD, coronary artery disease; CARDIoGRAMplusC4D, Coronary ARtery DIsease Genome wide Replication and Meta-analysis plus The Coronary Artery Disease Genetics; IBD, inflammatory bowel disease; ILCCO, International Lung Cancer Consortium; GWAS, genome-wide association study; MR, Mendelian randomization; OCAC, The Ovarian Cancer Association Consortium; PRACTICAL, Prostate Cancer Association Group to Investigate Cancer Associated Alterations in the Genome consortium; RA, rheumatoid arthritis; SNPs, single-nucleotide polymorphisms; TNF, tumour necrosis factor; VTE, venous thromboembolism.

**Fig. 2:**
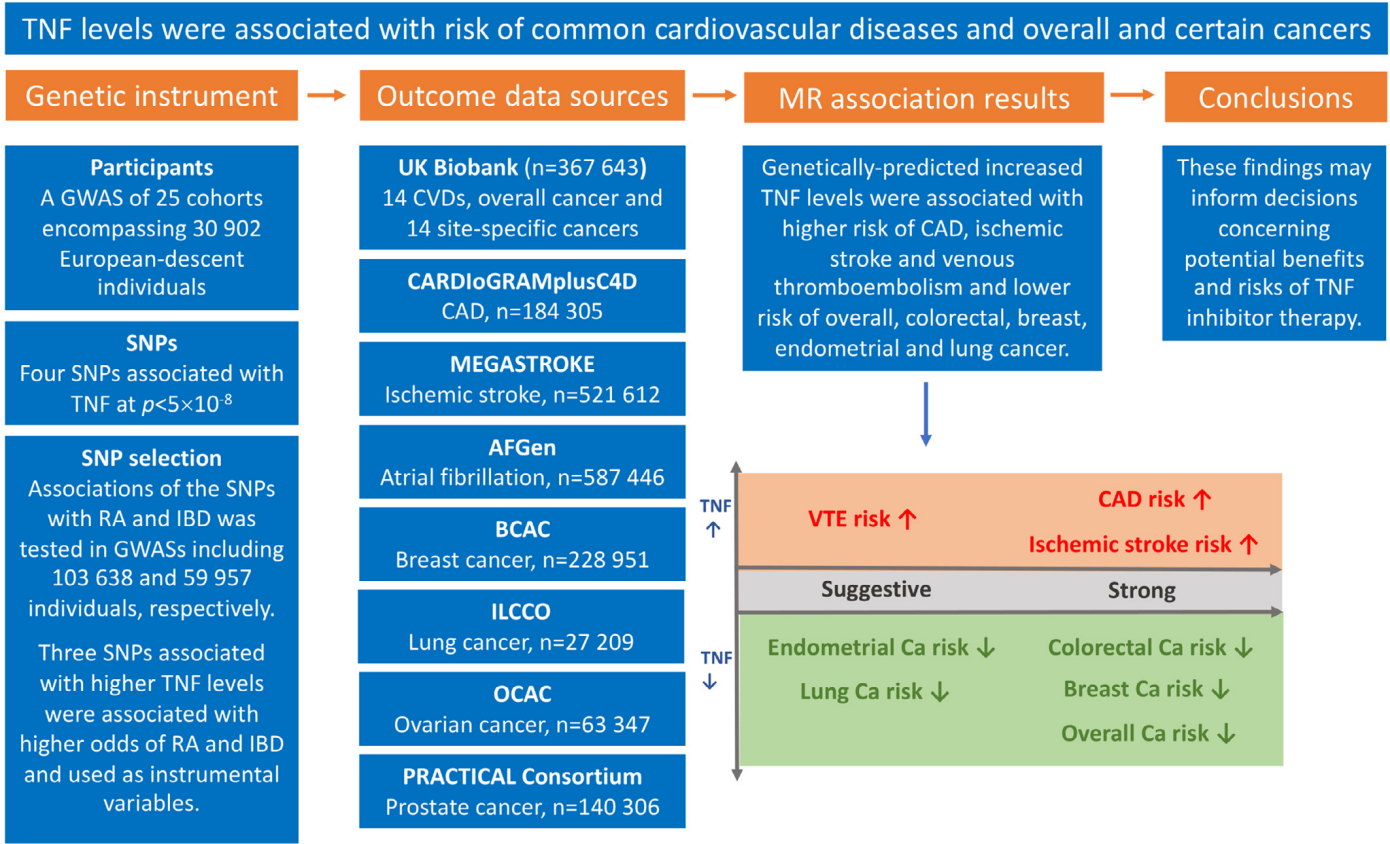
Associations of genetically higher TNF levels with cardiovascular diseases and cancers. AFGen indicates Atrial Fibrillation Consortium; BCAC, Breast Cancer Association Consortium; CARDIoGRAMplusC4D, Coronary ARtery DIsease Genome wide Replication and Meta-analysis plus The Coronary Artery Disease Genetics; CI, confidence interval; ILCCO, International Lung Cancer Consortium; NA, not available; OCAC, The Ovarian Cancer Association Consortium; OR, odds ratio; PRACTICAL, Prostate Cancer Association Group to Investigate Cancer Associated Alterations in the Genome consortium; TNF, tumour necrosis factor; UKBB, UK Biobank.

The associations of TNF levels instrumented by three SNPs with the CVD and cancer outcomes are displayed in Fig. 1 and Fig. 2. Genetically higher TNF levels were associated with higher odds of coronary artery disease and ischaemic stroke and lower odds of overall, colorectal, and breast cancer. For one unit increase in natural log-transformed TNF levels, the ORs were 2.25 (95% CI, 1.50, 3.37) for coronary artery disease, 2.27 (95% CI, 1.50, 3.43) for ischaemic stroke, 0.54 (95% CI, 0.42, 0.96) for overall cancer, 0.51 (95% CI, 0.39, 0.67) for breast cancer, and 0.20 (95% CI, 0.09, 0.45) for colorectal cancer. Results for coronary artery disease and breast cancer were similar in UK Biobank and consortia. There was weak evidence of association between TNF levels and ischaemic stroke in UK Biobank. Genetically predicted TNF levels showed a suggestive positive association with risk of venous thromboembolism (OR 2.18, 95% CI 1.32, 3.59) and inverse associations with risk of endometrial cancer (OR 0.25, 95% CI 0.07, 0.94) and lung cancer (OR 0.45, 95% CI 0.21, 0.94). Genetically predicted TNF levels were not associated with the other studied cardiovascular diseases and site-specific cancers in the main analysis. In the supplementary analysis, using four SNPs, there was some evidence of inverse associations of genetically-predicted TNF levels with intracerebral haemorrhage (OR, 0.19; 95% CI, 0.04, 0.92), colorectal cancer (OR, 0.23; 95% CI, 0.09, 0.60), and ovarian cancer (OR, 0.23; 95% CI, 0.06, 0.91) (**Supplementary figure 1**).

## 4 Discussion

In the present MR study, we provided the first causal evidence of positive associations of TNF levels with atherothrombotic disease (coronary artery disease and ischaemic stroke) and venous thromboembolism. Furthermore, we revealed inverse associations of TNF levels with risk of overall cancer and several site-specific cancers (colorectal, breast, endometrial, and lung cancers). We confirmed that higher TNF levels were strongly associated with established TNF-driven diseases (rheumatoid arthritis and inflammatory bowel disease) which added strong support to the validity of the genetic instrument used and the reliability of our findings.

### 4.1 Primary findings in cardiovascular disease

A correlation between a TNF-related SNP and CVD was found in a study with 587 patients, which showed an association between the TNFA rs1800629 gene variant and cardiovascular complications in patients with rheumatoid arthritis albeit confined within individuals carrying the rheumatoid shared epitope [25]. The present study comprehensively examined associations of TNF levels with most common CVDs amongst a general population and revealed positive associations of TNF levels with atherothrombotic disease and venous thromboembolism.

Atherothrombotic disease is a chronic inflammatory disease of the arterial wall and has been shown to be TNF-driven. A possible positive association of TNF with ischaemic stroke [26] has been reported. In addition, TNF inhibition in patients with rheumatoid arthritis improves important correlates of CVD such as carotid intimal-medial thickness and aortic stiffness [27], and has been shown to reduce the risk of overall cardiovascular events [28], myocardial infarction and stroke in rheumatoid arthritis patients [6]. However, the putative role of TNF in driving this may differ in the general population and patients with inflammatory arthropathies subject to systemic inflammation, medications known to drive CVD such as nonsteroidal anti-inflammatory drugs and steroids and more abundant traditional risk factors. Our findings support previous research for TNF driving atherothrombosis and extend it to the general population. Importantly, targeting inflammation using the biological therapy has previously been successful. Canakinumab, which neutralizes IL1B, reduced recurrent cardiovascular outcomes in patients with a high inflammatory burden in a clinical trial even though the results of this trial were substantially lower than expected [29]. The underlying mechanism for TNF-driven atherothrombosis could be via a variety of proposed mechanisms, including favourable effects on circul1ating lipids, insulin resistance, endothelial dysfunction, leucocyte recruitment, oxidative stress, vasodilation or coagulation [30]. The observed positive association of genetically-predicted TNF levels with venous thromboembolism is not found in traditional observational studies showing no association [31,32], but the precision was low in those studies. However, a recent longitudinal cohort study based on the German register RABBIT revealed that anti-TNF agents decreased the risk of serious venous thromboembolism events compared to csDMARDs medicine [33], which is in line with our finding. Venous thromboembolism differs in pathology from arterial, which is driven by the atherosclerotic process. Even though inflammation and the innate immune system have an important role in venous thromboembolism, the link between TNF and thrombogenesis remains unclear. On one hand, TNF has been proposed to promote a pro-coagulant state. On the other hand, a recent study in mice found an essential role in the resolution of venous thrombus through the TNF receptor (TNF-Rp55) in intrathrombotic macrophages with no effect on coagulation [34].

### 4.2 Primary findings in cancer

With regard to overall cancer risk, randomized controlled trials and observational studies assessing the effect of TNF inhibitor treatment, primarily in rheumatoid arthritis and inflammatory bowel disease patients, have yielded inconclusive results [35,36]. This may relate to the complexities of such studies with rare cancer outcomes, short follow-up, high patient exclusions and the potential of reverse causation with the neoplastic process itself affecting levels of inflammatory mediators. Furthermore, there may be confounding from the underlying inflammatory disease or concomitant treatments such as non-steroidal anti-inflammatory drugs or disease-modifying anti-rheumatic drugs. Both a study with a long 10-year follow up [36], and a large meta-analysis of 6 randomized controlled trials [37] demonstrated it, although the latter themselves have been reported to increase cancer risk themselves [37]. The present MR study found an inverse association between TNF levels and overall cancer in UK Biobank, but we cannot exclude that the observed association might be driven by several site-specific cancers contributing a large proportion of cancer cases, such as breast cancer (18%) and colorectal cancer (7%). In any case, our MR study, which avoids many of the aforementioned limitations of previous studies, provides evidence that anti-TNF therapies may promote the development of some cancer types.

Previous studies of TNF levels in relation to risk of colorectal cancer are inconsistent. Carcinogenic effects have been suggested by a clinical study of 30 colorectal cancer patients in which the TNF gene was significantly overexpressed in cancerous tissue compared with adjacent normal colorectal tissue [38]. Although this does not provide causal evidence, in genetic studies polymorphisms of TNF have been associated with colorectal cancer risk [39]. Conversely, register-based studies have detected both no difference [36], and, an increased colorectal cancer risk [40], compared to untreated patients. Epidemiological data on TNF levels in relation to risk of breast, lung and endometrial cancer are also conflicting and scarce. A genetic study showed that *TNFA−308* A allele was associated with a lower risk of breast cancer amongst European populations [41]. However, several register-based and cohort studies have found no association between TNF levels and breast cancer risk [42] or a reduced risk of breast cancer with TNF inhibitor use [36]. The protective effect of anti-TNF therapy observed on breast cancer risk may have been confounded by unmeasured effects of non-steroidal anti-inflammatory drugs [43] and other synthetic disease-modifying anti-rheumatic drugs [44] in patients with autoimmune diseases. Endometrial cancer risk was increased in women with elevated pre-diagnostic concentrations of TNF in a case-control study with 270 cases and 518 controls [45]. However, there was no association between TNF and endometrial cancer in a prospective study [46].

### 4.3 Clinical implications

Increased risk of cardiovascular disease with genetically predicted high TNF level sheds light on the usage of anti-TNF medicine as a potential prevention approach for people with excessive risk of CVD and a potential treatment strategy for patients with impaired cardiovascular condition. In addition, clinicians need to assess the potential increased CVD risk derived from TNF therapy especially amongst individuals with inherited or acquired high risk of CVD. With regard to the observed protective effect of TNF on cancer, our study reveals two important clinical considerations. Firstly, it suggests recombinant TNF therapy as a potential therapy in such cancers, in particular colorectal and breast cancers. Phase 2 trials of recombinant TNF across a range of cancer types have so far not proven successful in causing tumour responses [47] and associated with significant toxicity [48]. An exception is the use of local TNF administered locally by isolated limb perfusion treatments in melanoma and sarcoma [47], or in isolated hepatic perfusion for treatment of liver metastasis [49] which have demonstrated that TNF alone or in combination to cause large response rates of up to 80%. Such studies have been focused on advanced and metastatic cancers for which prognoses are poor and a significant tumour response would be unlikely. Future studies should assess the tumour responses in patients with earlier-staged disease and in combination therapies. The second important clinical implication of the inverse associations observed between TNF levels and cancer risk relates to the use of anti-TNF biological therapies, which are highly effective and ingrained in guidelines for the management of conditions such as rheumatoid arthritis and inflammatory bowel disease. Such therapies have previously been associated with concerns regarding cancer risk, particularly lymphoma [50] and non-melanoma skin cancer [51]. In line with the general consensus, we do not demonstrate a significant association with these cancer types.

### 4.4 Strengths, limitations and caveats

The MR design, which diminishes confounding and reverse causality, was the major strength of this study. Additionally, we comprehensively assessed the causal associations of TNF levels with a broad range of CVD and cancer outcomes. Data were mainly extracted from individuals of European ancestry, except for a few outcomes with a small portion of individuals of non-European ancestry. Moreover, the SNP-exposure and SNP-outcome estimates were adjusted for principal components for ancestry. Thus, population stratification bias is unlikely to have had an essential effect on our results. On the other hand, this population confinement, on a certain degree, compromised the generalizability of the study results to other populations, such as Asians, African Americans, etc. A major limitation is that the number of cases was few for some CVDs and site-specific cancers, which resulted in low precision of the estimates. Thus, we may have missed weak associations.

The results of this MR study should be interpreted in light of the pleiotropic effects of TNF, which plays a role in a wide range of biological processes, such as immunity, inflammation, apoptosis, lipid metabolism, and coagulation [1], [2], [3]. Although the observed associations of genetically higher TNF levels with increased risk of CVD (particularly atherosclerotic- and thrombotic-related CVDs) and lower risk of cancer are biologically plausible, we cannot entirely rule out that our results might have been affected by horizontal pleiotropy. For example, three of the four SNPs were associated with hypothyroidism, potentially reflecting autoimmune thyroiditis. The possible role of hypothyroidism in mediating (vertical pleiotropy) or biasing (horizontal pleiotropy) the results are unclear. In addition, even though the instrumental variables used were validated using two inflammatory diseases as positive controls, our findings need to be interpreted with caution given that the excluded SNP may have influences on inflammation in an opposite pathway or atherosclerosis only. Based on current findings, a comparative effect on cardiovascular system and carcinogenesis of anti-TNF therapies and treatments established on other biological mechanisms cannot be determined. Thus, the study provides limited evidence on drug selection in rheumatic disease treatment. Considering high risk of certain malignancies in individuals with rheumatic disease [52], randomized controlled trials are warranted to verify our findings and comprehensively evaluate the benefits and risks of anti-TNF therapy in populations with different health conditions, even though TNF levels of most included participants were in the healthy range [12].

## Conclusions

This MR study found evidence of causal associations of increased TNF levels with higher risk of coronary artery disease, ischaemic stroke, and venous thromboembolism and decreased risk of overall, colorectal, breast, endometrial, and lung cancer. Along with previous observational studies [6,10], the present study strengthened the evidence that TNF inhibitors might reduce the risk of common cardiovascular events but increase risk of overall and certain cancers. These results may inform decisions concerning potential benefits and risks of TNF inhibitor therapy.

## Author contributions

Study design: S.Y., P.C., M.B., M.V., S.K., A.M.M., A.L., S.B., S.C.L.; data acquisition and analysis: S.Y., A.M.M., S.B., S.C.L.; figures and writing S.Y.; reviewing and editing: S.Y., P.C., M.B., M.V., S.K., A.M.M., A.L., A.L., S.B., S.C.L.

## Declaration of Competing Interests

SCL reports grants from Swedish Research Council (Vetenskapsrådet; grant no. 2019-00977), grants from Swedish Research Council for Health, Working Life and Welfare (Forte; grant no. 2018-00123), grants from Swedish Heart-Lung Foundation (Hjärt-Lungfonden; grant no. 20190247), during the conduct of the study. AMM reports grants from EC-Innovative Medicines Initiative (BigData@Heart), during the conduct of the study; .All other authors declare no conflicts of interest.

## Appendix Supplementary materials

Image, application 1
