# Supplementary material for: Effects of tumour necrosis factor on cardiovascular disease and cancer: A two-sample Mendelian randomization study
Source: eBioMedicine. 2020 Aug 14;59:102956. doi: 10.1016/j.ebiom.2020.102956 (PMC7452586; doi:10.1016/j.ebiom.2020.102956)
Supplement: Supplementary file 1 [file mmc1.docx]

**SUPPLEMENTARY MATERIAL**

**Effects of tumor necrosis factor on cardiovascular disease and cancer: a two-sample Mendelian randomization study**

Shuai Yuan, MD^1,2^, Paul Carter, MBChD^3^, Maria Bruzelius, MD, PhD^4,5^; Mathew Vithayathil, MBBS^6^, Siddhartha Kar, MBBS^7^, Amy M. Mason, PhD^8,9^, Ang Lin, PhD^10,11^, Stephen Burgess, PhD^3,10^, Susanna C. Larsson, PhD^1,2^

^1^ Unit of Cardiovascular and Nutritional Epidemiology, Institute of Environmental Medicine, Karolinska Institutet, Stockholm, Sweden

^2^ Department of Surgical Sciences, Uppsala University, Uppsala, Sweden

^3^ Department of Public Health and Primary Care, University of Cambridge, Cambridge, UK

^4^ Coagulation Unit, Department of Hematology, Karolinska University Hospital, Stockholm, Sweden

^5^ Department of Medicine Solna, Karolinska Institutet, Stockholm, Sweden

^6^ MRC Cancer Unit, University of Cambridge, Cambridge, UK

^7^ MRC Integrative Epidemiology Unit, Bristol Medical School, University of Bristol, Bristol, UK

^8^ British Heart Foundation Cardiovascular Epidemiology Unit, Department of Public Health and Primary Care, University of Cambridge, Cambridge, UK

^9^ National Institute for Health Research Cambridge Biomedical Research Centre, University of Cambridge and Cambridge University Hospitals, Cambridge, UK

^10^ Department of Medicine Solna, Division of Immunology and Allergy, Karolinska Institutet, Stockholm, Sweden

^11^ Center for Molecular Medicine, Karolinska Institutet, Stockholm, Sweden

^10^ MRC Biostatistics Unit, University of Cambridge, Cambridge, UK

**Supplementary table 1.** Associations of genetically predicted higher TNF levels with rheumatoid arthritis and inflammatory bowel disease in analysis including all four SNPs and in leave-one-out analysis excluding one SNP at a time

|  | Rheumatoid arthritis | | |  | | | Inflammatory bowel disease | | |
| --- | --- | --- | --- | --- | --- | --- | --- | --- | --- |
| Excluded SNP (Nearby gene) | OR | 95% CI | P | |  | OR | | 95% CI | P |
| None; including all four SNPs | 0.54 | 0.03, 10.9 | 0.686 | |  | 0.65 | | 0.03, 14.4 | 0.784 |
| rs2857602 (*LTA*) | 2.25 | 1.06, 4.78 | 0.036 | |  | 3.02 | | 1.42, 6.41 | 0.004 |
| rs10744774 (*BRAP*) | 0.45 | 0.01, 17.5 | 0.671 | |  | 0.58 | | 0.01, 24.7 | 0.778 |
| rs3184504 (*SH2B3*) | 0.32 | 0.02, 5.46 | 0.435 | |  | 0.41 | | 0.02, 8.08 | 0.558 |
| rs7182229 (*LIPC*) | 0.45 | 0.22, 0.93 | 0.030 | |  | 0.45 | | 0.22, 0.93 | 0.030 |

CI, confidence interval; OR, odds ratio; SNP, single nucleotide polymorphism; TNF, tumor necrosis factor.

Rs7182229 was not available in the dataset of inflammatory bowel disease. A proxy SNP (rs11631312, R^2^=0.92 in CEU

population) was used in all analysis that included rs7182229.

There is substantial significant heterogeneity in all analyses, except in the analyses of excluding rs2857602.

**Supplementary table 2.** Possible pleiotropic associations of the instrumental variables for TNF with other phenotypes at genome-wide significance

| **SNP (Gene)** | **EA** | **Phenotype** | | | | | | |
| --- | --- | --- | --- | --- | --- | --- | --- | --- |
| rs2857602 (*LTA*)* | G | Celiac disease (-) | Hypothyroidism (-) | Treatment with insulin product (-) | Rheumatoid arthritis (-) | Monocyte (+) | Hemoglobin  (+) | Mouth ulcers (-) |
|  |  | Height (+) | Red cell distribution width (-) | Eosinophil (-) | Type 1 diabetes (-) | Forced vital capacity (+) | IgA deficiency (-) |  |
| rs10744774 (*BRAP*) | A | Plateletcrit (+) | Eosinophil count (+) | Neutrophil percentage of granulocytes (-) | Lymphocyte count (+) | Hypothyroidism (+) | Diastolic blood pressure (+) | White blood cell count (+) |
|  |  | Hemoglobin concentration (+) | Red blood cell count (+) | Reticulocyte count (+) |  |  |  |  |
| rs3184504 (*SH2B3*) | T | Plateletcrit (NA) | Eosinophil count (+) | Lymphocyte count (NA) | Hypothyroidism (+) | Hemoglobin concentration (+) | White blood cell count (-) | Diastolic blood pressure (+) |
|  |  | Type 1 diabetes (NA) | Coronary artery disease (+) | Self-reported hypertension (+) | Celiac disease (NA) | Total cholesterol (-) | Rheumatoid arthritis (+) | LDL cholesterol (NA) |
|  |  | Myocardial infarction (NA) | Fibrinogen levels (NA) | Hip circumference (-) | Height (-) | Weight (-) | Colorectal cancer (-) | Beta 2 microglubulin plasma levels (-) |
| rs7182229 (*LIPC*) | T | None |  |  |  |  |  |  |

EA indicates effect allele (the allele associated with higher TNF-ɑ levels); NA, not available; SNP, single-nucleotide polymorphism. +/- means positive/inverse associations between effect allele and phenotypes. *This variant was not included in the primary analyses.

**Supplementary table 3.** Power calculations for the Mendelian randomization analyses performed in the current study

| **Outcome** | **Source** | **Cases** | **Sample size** | **% cases** | **Detectable OR* at 80% power** |
| --- | --- | --- | --- | --- | --- |
| **Inflammatory disease** |  |  |  |  |  |
| Rheumatoid arthritis | GARNET consortium | 29880 | 103638 | 28.83 | ≤0.76 or ≥1.27 |
| Inflammatory bowel disease | UK IBD consortium | 25042 | 59957 | 41.77 | ≤0.73 or ≥1.35 |
| **Cardiovascular disease** |  |  |  |  |  |
| Cerebrovascular disease |  |  |  |  |  |
| Overall stroke | MEGASTROKE consortium | 67162 | 521612 | 12.88 | ≤0.85 or ≥1.16 |
| Overall stroke | UKBB | 9652 | 367643 | 2.63 | ≤0.63 or ≥1.37 |
| Any ischemic stroke | MEGASTROKE consortium | 60341 | NA | NA | NA |
| Any ischemic stroke | UKBB | 3554 | 367643 | 0.97 | ≤0.39 or ≥1.61 |
| Large artery stroke | MEGASTROKE consortium | 6688 | 153080 | 4.37 | ≤0.55 or ≥1.47 |
| Small vessel stroke | MEGASTROKE consortium | 11710 | 204372 | 5.73 | ≤0.65 or ≥1.36 |
| Cardioembolic stroke | MEGASTROKE consortium | 9006 | 213576 | 4.22 | ≤0.61 or ≥1.40 |
| Intracerebral hemorrhage | UKBB | 1064 | 367643 | 0.29 | ≤0.01 or ≥2.11 |
| Subarachnoid hemorrhage | UKBB | 1084 | 367643 | 0.29 | ≤0.01 or ≥2.11 |
| Heart disease |  |  |  |  |  |
| Coronary artery disease | CARDIoGRAMplusC4D consortium | 60801 | 184305 | 32.99 | ≤0.82 or ≥1.20 |
| Coronary artery disease | UKBB | 24531 | 367643 | 6.67 | ≤0.76 or ≥1.25 |
| Heart failure | UKBB | 7382 | 395034 | 1.87 | ≤0.57 or ≥1.43 |
| Atrial fibrillation | AFGen | 65446 | 587446 | 11.14 | ≤0.85 or ≥1.16 |
| Atrial fibrillation | UKBB | 16945 | 367643 | 4.61 | ≤0.71 or ≥1.30 |
| Abdominal aortic aneurysm | UKBB | 1094 | 367643 | 0.30 | ≤0.01 or ≥2.11 |
| Aortic valve stenosis | UKBB | 2244 | 367643 | 0.61 | ≤0.22 or ≥1.79 |
| Vessel disease |  |  |  |  |  |
| Peripheral artery disease | UKBB | 3415 | 367643 | 0.93 | ≤0.38 or ≥1.62 |
| Venous thromboembolism | UKBB | 15602 | 367643 | 4.24 | ≤0.71 or ≥1.30 |
| **Cancer** |  |  |  |  |  |
| Bladder cancer | UKBB | 2588 | 367643 | 0.70 | ≤0.28 or ≥1.73 |
| Breast cancer | BCAC | 122977 | 228951 | 53.71 | ≤0.86 or ≥1.17 |
| Breast cancer ER- | BCAC | 21468 | NA | NA | NA |
| Breast cancer ER+ | BCAC | 69501 | NA | NA | NA |
| Breast cancer | UKBB | 13666 | 198838 | 6.87 | ≤0.68 or ≥1.33 |
| Cervical cancer | UKBB | 1928 | 198838 | 0.97 | ≤0.14 or ≥1.86 |
| Colorectal cancer | UKBB | 5486 | 367643 | 1.49 | ≤0.50 or ≥1.50 |
| Endometrial cancer | UKBB | 1520 | 198838 | 0.76 | 0.05≤ or ≥1.96 |
| Head-neck cancer | UKBB | 1615 | 367643 | 0.44 | 0.05≤ or ≥1.97 |
| Kidney cancer | UKBB | 1310 | 367643 | 0.36 | 0.05≤ or ≥1.98 |
| Leukemia | UKBB | 1403 | 367643 | 0.38 | 0.05≤ or ≥1.99 |
| Lung cancer | ILCCO | 11348 | 27209 | 41.71 | ≤0.62 or ≥1.55 |
| Melanoma | UKBB | 4869 | 367643 | 1.32 | ≤0.47 or ≥1.54 |
| Non-Hodgkin's lymphoma | UKBB | 2296 | 367643 | 0.62 | ≤0.22 or ≥1.79 |
| Ovarian cancer | UKBB | 1520 | 198838 | 0.76 | 0.05≤ or ≥1.99 |
| Ovarian cancer | OCAC | 22 406 | 63 347 | 0.35 | 0.67≤ or ≥1.43 |
| Overall cancer | UKBB | 75037 | 367643 | 20.41 | ≤0.85 or ≥1.16 |
| Pancreatic cancer | UKBB | 1264 | 367643 | 0.34 | ≤0.01 or ≥2.11 |
| Prostate cancer | PRACTICAL | 79194 | 140306 | 56.44 | ≤0.82 or ≥1.23 |
| Prostate cancer | UKBB | 7872 | 168748 | 4.66 | ≤0.58 or ≥1.43 |

Power calculation were based on the online application “mRnd: Power calculations for Mendelian Randomization” (<http://cnsgenomics.com/shiny/mRnd/>).

Variance explained by 4 selected TNF-ɑ related SNP was 0.58%.

AFGen indicates Atrial Fibrillation Consortium; BCAC, Breast Cancer Association Consortium; CARDIoGRAMplusC4D, Coronary ARtery DIsease Genome wide Replication and Meta-analysis plus The Coronary Artery Disease Genetics; GARNET, Genetics and Allied research in Rheumatic diseases Networking; ILCCO, The International Lung Cancer Consortium; NA, not available; OCAC, The Ovarian Cancer Association Consortium; PRACTICAL, The Prostate Cancer Association Group to Investigate Cancer Associated Alterations in the Genome consortium; UKBB, UK Biobank; UK IBD consortium, UK Inflammatory Bowel Disease Genetics Consortium. *Includes Crohn's disease and ulcerative colitis.

**Supplementary figure 1.** Associations of genetically predicted high TNF levels with cancers and cardiovascular diseases using 4 SNPs

**
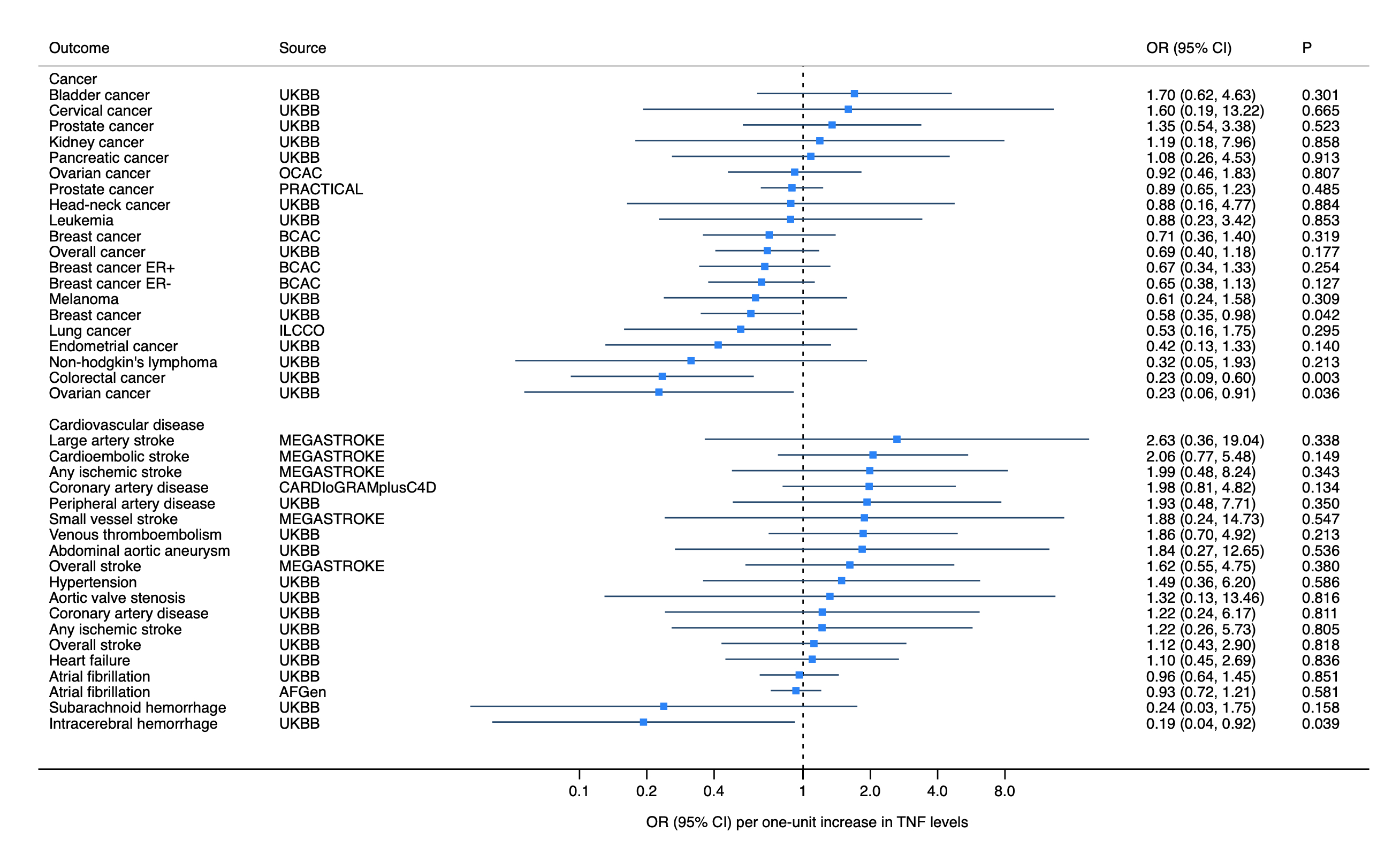
**

AFGen indicates Atrial Fibrillation Consortium; BCAC, Breast Cancer Association Consortium; CARDIoGRAMplusC4D, Coronary ARtery DIsease Genome wide Replication and Meta-analysis plus The Coronary Artery Disease Genetics; CI, confidence interval; ILCCO, International Lung Cancer Consortium; NA, not available; OCAC, The Ovarian Cancer Association Consortium; OR, odds ratio; PRACTICAL, Prostate Cancer Association Group to Investigate Cancer Associated Alterations in the Genome consortium; TNF, tumor necrosis factor; UKBB, UK Biobank.
